# Low Birth Weight, Cumulative Obesity Dose, and the Risk of Incident Type 2 Diabetes

**DOI:** 10.1155/2018/8435762

**Published:** 2018-01-09

**Authors:** Cindy Feng, Nathaniel D. Osgood, Roland F. Dyck

**Affiliations:** ^1^School of Public Health, University of Saskatchewan, Saskatoon, SK, Canada; ^2^Department of Computer Science, University of Saskatchewan, Saskatoon, SK, Canada; ^3^Department of Medicine, Canadian Centre for Health and Safety in Agriculture, University of Saskatchewan, Saskatoon, SK, Canada

## Abstract

**Background:**

Obesity history may provide a better understanding of the contribution of obesity to T2DM risk.

**Methods:**

17,634 participants from the 1958 National Child Development Study were followed from birth to 50 years. Cumulative obesity dose, a measure of obesity history, was calculated by subtracting the upper cut-off of the normal BMI from the actual BMI at each follow-up and summing the areas under the obesity dose curve. Hazard ratios (HRs) for diabetes were calculated using Cox regression analysis. Three separate models compared the predictive ability of cumulative obesity dose on diabetes risk with the time-varying BMI and last BMI.

**Results:**

In final models, 341 of 15,043 (2.27%) participants developed diabetes; male sex and low birth weight were significant confounding variables. Adjusted HRs were 1.080 (95% CI: 1.071, 1.088) per 10-unit cumulative obesity dose, 1.098 (95% CI: 1.080, 1.117) per unit of the time-varying BMI, and 1.146 (95% CI: 1.084, 1.212) per unit of the last BMI. Cumulative obesity dose provided the best predictive ability for diabetes.

**Conclusions:**

Cumulative obesity dose is an improved method for evaluating the impact of obesity history on diabetes risk. The link between low birth weight and T2DM is strengthened by adjusting for cumulative obesity dose.

## 1. Introduction

A pandemic of type 2 diabetes (T2DM) is affecting diverse populations worldwide [1] and is strongly linked to global increases in rates of overweight and obesity which have risen dramatically over the past several decades [2]. While the immediate relationship between increased adiposity and T2DM is firmly established [1, 2], emerging evidence suggests that obesity duration and its age of onset (obesity history) may provide greater insight into the contribution of obesity to T2DM risk than into cross-sectional relationships alone [3–11]. Moreover, while there is also an established link between both low birth weight and high birth weight and the development of T2DM [12], it is not clear if and how birth weight contributes to higher diabetes risk through its possible impact on the onset, degree, and duration of excess weight.

Several approaches have been used to evaluate the relationship between obesity history and subsequent morbidity/mortality, but models incorporating a duration-of-obesity component have performed most strongly [13]. One such model type combines time interval with a measure of the degree to which an individual's body mass index (BMI) exceeds either a normal BMI or some other BMI baseline [13]. This has been described as being akin to pack years of smoking [4], and different versions of this methodology have all shown a significant correlation between an increasing area under the excess BMI curve and diabetes risk [14–17]. However, the four main studies that have used this approach [14–17] have limitations with the absence of data from the younger period of the life cycle, short study duration, the manner in which the excess BMI was calculated, or a combination of those and other drawbacks. Importantly, none of these studies have included birth weight as a potential confounder.

Using a British birth cohort from the 1958 National Child Development Study (NCDS) [18], we now report on the largest and longest study to date that has examined the relationship between the duration-of-obesity measure and T2DM risk. By following individuals from birth to age 50, our primary objective was to determine the influence of what we believe is an improved method of calculating the duration of obesity—what we refer to as cumulative obesity dose—on the incidence of diabetes, while adjusting for birth weight as well as for other known diabetes risk factors. Our secondary objective was to compare the strength of this relationship with the performance of the time-varying BMI and most recent BMI, in predicting diabetes risk. Deciphering the impact and dynamics of cumulative obesity dose on diabetes risk, while taking into account the influence of birth weight, could help us to better understand the emergence of type 2 diabetes, to guide in the timing and the nature of public health measures designed to curb the epidemics of both obesity and T2DM, and finally to provide a more informed basis on which to provide individual-level obesity counselling.

## 2. Methods

### 2.1. Study Population

The participants were from the National Child Development Study [18], an ongoing longitudinal study of a cohort of 17,634 children born in England, Wales, and Scotland during the week of March 9, 1958. To date, there have been eight follow-up sweeps that have traced the members of the cohort throughout childhood (aged 7, 11, and 16 years) and into adulthood (aged 23, 33, 42, 46, and 50 years). The main sample of the present study included 16,573 who had had at least two follow-ups. Written informed consent was obtained from the participants, and ethical approval for the study was obtained from the South East Multi-Centre Research Ethics Committee.

### 2.2. The Main Outcome Measure—Incidence of Diabetes

The occurrence of diabetes was determined by a self-reported response to the question, “Have you ever been diagnosed with diabetes?” at each sweep and by using information from sweep 6, when the 42-year-old participants were asked: “At what age were you diagnosed with diabetes?” No information was provided about the diabetes type, and no medical verification of diagnosis was available. While it is possible that T2DM may have been underdiagnosed among youth and young adults in the 1970s, because of the long follow-up period of this study, these individuals would likely have been diagnosed at a later age.

The self-reported age of diabetes diagnosis for those participants who remained in the study at age 42 was used as the diabetes incidence age for those individuals. For those who dropped out of the study before age 42 or who developed diabetes after sweep 6, the incidence age of diabetes diagnosis was estimated as the midpoint between the last diabetes-free follow-up and the follow-up visit when diabetes was first reported. Participants were censored at the age of their last follow-up visit if they remained diabetes-free at that time.

### 2.3. Key Risk Factors of Interest—Body Mass Index and Cumulative Obesity Dose

For each sweep, we calculated the BMI based on participants' weight and height (BMI = weight in kg/height in m^2^). Height was not reported at sweeps 7 and 8, so height at sweep 6 was used as a proxy for later sweeps. To account for missing BMI values, we used the predicted values by fitting a generalized linear mixed-effects model on the logarithm of the BMI with age and sex as predictors. To calculate the degree of obesity dose, we first subtracted the reference BMI from the actual BMI. For the purposes of this study, the reference BMI was defined as the upper cut-off for normal BMIs which varies with age and sex. The cut-off values were 17.9 kg/m^2^ for boys and 17.8 kg/m^2^ for girls at age 7, 20.6 kg/m^2^ for boys and 20.7 kg/m^2^ for girls at age 11, 23.9 kg/m^2^ for boys and 24.4 kg/m^2^ for girls at age 16, and 25 kg/m^2^ for both genders at age 23 and above [19]. If the difference was negative, it was treated as zero. The cumulative obesity dose takes into account the duration and degree of obesity and was calculated by summing the areas under the obesity dose curve over all previous years beginning at age 7 up to the current sweep. For ease of computation, we calculated the area numerically. Cumulative obesity dose was modeled as a time-dependent variable in the analysis, which was presented as multiple observations for a single subject, each of which applied to a time interval of observation.

The time-varying BMI [20] was determined by the repeated measurements of the BMI for a subject, which may change over the period of time that the subject is observed. The follow-up time for each patient was divided into different time windows. For each time window, a separate Cox analysis was carried out using the time-dependent variable at the beginning of that specific time window. Then, a weighted average of all the time window-specific results was calculated as the estimated relative ratio (please see Appendix A for a more detailed description).

The last BMI was the most recent BMI calculated before the diagnosis of diabetes. For those without diabetes, the last BMI was the BMI calculated at age 50 for those who remained in the study at sweep 8 or the final BMI calculated for those who dropped out of the study before age 50.

### 2.4. Measurement of Other Diabetes Predictors

The age of the participants was defined as the years from the date of birth to the last interview year. Ethnicity was grouped into Caucasian versus others. The participants' birth weights were grouped as low (less than 2.5 kg), normal (2.5–4.0 kg), and high (above 4.0 kg). The baseline social class of each participant's mother's husband was used as a proxy for the socioeconomic status of the participant. These were categorized as I (professional occupations), II (managerial and technical occupations), III (skilled occupations), IV (partly skilled occupations), and V (unskilled occupations).

### 2.5. Statistical Analysis

Potential confounding variables that may be related to the BMI and onset of diabetes and that were included in the analysis were ethnicity, sex, participants' birth weight, and social class. The univariate relationships between the potential confounding variables and incidence of diabetes were expressed as hazard ratios (HRs) and determined using Cox proportional hazard regression analysis. In multivariate analysis, the effects of cumulative obesity dose, time-varying BMI, and last BMI were examined as time-dependent covariates in separate models using Cox proportional hazard modeling, with the onset of diabetes as the dependent variable. We also included a model without measures of obesity with which to compare the others. Each of the models was adjusted for potential confounding variables that were significant at the 0.2 level of significance in the univariate analysis and were modeled as fixed (non-time-dependent) covariates. In the final multivariate models, backward model selection was carried out and the final models only included covariates that were significant at the 0.05 level of significance.

To study the specific effect of age of obesity onset on the risk of developing diabetes, while adjusting for the effect of cumulative obesity dose, a subset analysis using multivariate Cox regression analysis was carried out. This was restricted to those participants who had developed overweight/obesity during the follow-up because no age of overweight/obesity onset can be determined for those who had never experienced it. In this analysis, covariate effects were assumed to be constant over time.

Finally, to study if the effect of cumulative obesity dose on diabetes risk varies by age groups, we evaluated the interaction between cumulative obesity dose and the three age groups (7–16, 23–42, and older than 42) in the model.

The Akaike information criterion (AIC) and Bayesian information criterion (BIC) were used to select the preferred model which was the one with the lowest AIC and BIC scores. All statistical analyses were performed using SAS software version 9.4 (SAS, Cary, NC, USA). All tests of significance were two-sided.

## 3. Results

The total number of participants who reported their diabetes status, the number of people newly diagnosed with diabetes, the number of people with an elevated BMI, and the total number of participants who were lost to follow-up at each sweep are shown in Table 1. Overall, 373 out of 16,573 (2.25%) participants reported their onset of diabetes. Although the proportion of participants who had an elevated BMI was relatively constant among children and youths, there was a progressive increase in those with an elevated BMI from age 23 on, and this increase was greater among men. By sweep 8, 13,655 (82.4%) of the initial cohort had been lost to follow-up.

The univariate relationships between the potential confounding variables and onset of diabetes are shown in Table 2. Ethnicity was borderline significant, with Caucasians displaying a lower hazard for diabetes than other groups. Males had a significantly higher risk of developing diabetes compared with females (HR 1.389; 95% CI: 1.131, 1.704). The association between birth weight of participants and the hazard of developing diabetes was borderline significant for low birth weight but was not significant for high birth weight. Participants from the lower social classes were at a significantly increased risk of developing diabetes. HR for class III versus class I was 1.963 (95% CI: 1.007, 3.825), for class IV versus class I was 2.159 (95% CI: 1.058, 4.406), and for class V versus class I was 2.543 (95% CI: 1.227, 5.271). The cumulative obesity dose, time-varying BMI, and last BMI were all significantly associated with an increased risk of diabetes onset. The unadjusted HR was 1.076 (95% CI: 1.068–1.084) for every 10-unit increase in cumulative obesity dose, the unadjusted HR was 1.098 (95% CI: 1.081–1.115) for every one-unit increase in the time-varying BMI, and the unadjusted HR was 1.137 (95% CI: 1.078–1.198) for the last BMI.

In the multivariate analysis (Table 3), 341 out of 15,043 (2.27%) participants developed diabetes during the study period after removing the participants with missing values in any of the covariates. All four final models contained participants' sex and birth weight as confounding variables. Ethnicity and social class did not remain in the final models after backward model selection. The adjusted HR (AHR) was 1.080 (95% CI: 1.071, 1.088) for every 10-unit increase in cumulative obesity dose, the AHR was 1.098 (95% CI: 1.080, 1.117) for every one-unit increase in the time-varying BMI, and the AHR was 1.146 (95% CI: 1.084, 1.212) for every one-unit increase in the last BMI. Finally, the AIC and BIC scores were much smaller for model number 1 with cumulative obesity dose as the covariate than the AIC and BIC scores for models number 2 and number 3, indicating that cumulative obesity dose provides much better predictive ability as compared to the time-varying BMI and last BMI.

Table 3 also shows the impact of birth weight on diabetes risk with and without the inclusion of measures of obesity. Without any of the measures of obesity in model number 0, the adjusted HR for low birth weight is very similar to the unadjusted HR in Table 2 (1.444 compared to 1.408) and remains not quite significant (*P* = 0.0732). In contrast, the AHRs for low birth weight are significant when any of the three measures of obesity are added in but particularly so for COD (1.686 for COD, 1.511 for the time-varying BMI, and 1.522 for the last BMI).

Table 4 shows the AHRs of developing diabetes among the 10,172/16,753 (61.38%) participants who developed overweight/obesity during follow-up. Among those, 310 (3.05%) developed diabetes. The three models all showed that older age of obesity onset is significantly associated with a decreased risk of developing diabetes, even after adjusting for other variables. Furthermore, male and low-birth weight participants who had a history of overweight/obesity were still at significantly higher risk of developing diabetes. Model number 1 still outperformed the other two models. However, in this subset analysis, the AIC and BIC results with and without age of obesity onset as a covariate showed that age of obesity onset improved the model fit to a greater degree for the time-varying BMI and last BMI than for cumulative obesity dose.

Table 5 shows that the effect of cumulative obesity dose on diabetes risk tends to decrease with older age groups. While the effect was not significant during childhood (possibly because of small numbers—AHR 1.187; CI 0.877, 1.607), it was higher for younger adults aged 23–42 (AHR 1.097; CI 1.081, 1.113) than for adults older than 42 (AHR slightly lower at 1.074; CI 1.064, 1.084). Nonetheless, there was no difference in AIC between this model and model number 1 in Table 3 indicating that the differential effect of cumulative obesity dose on diabetes risk across different age groups is minimal.

## 4. Discussion

Using the data from the 1958 National Child Development Study (NCDS) [18], we have now confirmed that combining a measure of excess weight with its duration from age 7 - cumulative obesity dose - is a strong and independent predictor of diabetes risk over the life course. We have additionally shown that cumulative obesity dose provides a greater predictive ability for diabetes risk than models incorporating either the time-varying BMI or last BMI before diabetes diagnosis. Thus, the larger the cumulative obesity dose, the greater the hazard for diabetes, with each increase of 10 units in cumulative obesity dose conferring an approximately 8% increase in diabetes risk. Moreover, as a function of obesity duration in particular, we found that the older the age of overweight/obesity onset, the less likely the individual is to develop diabetes. This suggests that, for a given amount of cumulative obesity dose, a longer duration of obesity is relatively (but only slightly) more important than the degree of obesity. The most novel finding of this study is that adjusting for cumulative obesity dose greatly strengthened the relationship between low birth weight and diabetes risk. Finally, although male sex independently added to the risk of diabetes, we were unable to demonstrate that ethnicity (small numbers) [1], social class [21], and high birth weight [22] contributed to diabetes risk in this population of predominantly white British subjects, as have been shown in other jurisdictions.

Longer duration and younger onset of obesity have both been shown to increase T2DM risk [3–11], but only recently has there emerged a relatively consistent approach in using obesity history to predict diabetes occurrence. Preston and colleagues described four main approaches that have been used to evaluate the links between obesity history and subsequent morbidity/mortality [13]. These include strictly additive models, duration-of-obesity models, additive weight change models, and interactive models. Models including a duration-of-obesity component performed most strongly. At least four groups have now reported on slightly differing versions of the duration-of-obesity model, which combines time interval with the measure of the degree to which an individual's BMI exceeds either a normal BMI or some other BMI baseline to forecast T2DM risk [14–17]. This measure has been likened to pack years of smoking [4]. However, all of these studies have limitations with the absence of data from the younger portion of the life cycle, short study duration, the way in which the excess BMI was calculated, or a combination of those and other drawbacks.

Using the data from the National Longitudinal Study of Youth, the first major study (2012) [14] using a duration-of-obesity methodology calculated what was termed “excess BMI-years” by subtracting a reference BMI of 25 (or the 85th BMI percentile for adolescents) from participants' BMIs, averaging the differences between survey years, and determining the total excess BMI-years by multiplying average differences by the study duration. Lee et al. found that a greater absolute number of excess BMI-years was significantly associated with higher T2DM risk and the risk was amplified by longer duration (i.e., those who experienced excess BMIs at younger ages). The limitation to the study is that the 8446 participants were enrolled between ages 14 and 21 years, thus missing the possible impact of childhood obesity and high/low birth weight. Moreover, it only followed participants for a total of 27 years when participants were aged 41–48. The second study (2013) [15] followed 1026 Framingham Offspring Study participants from their midthirties to midfifties using “cumulative excess weight” (CEW) as the outcome. CEW was calculated in a similar fashion to excess BMI-years (above) but was only associated with T2DM incidence for those with a normal BMI (<25) at baseline. Once again, the study was limited by a relatively short duration and an even larger omission of data from birth to early adulthood. Also, both of these studies in effect considered participants' BMIs below 25 as reducing diabetes risk since lower BMIs created a negative balance in their calculations.

Two more recent studies that have reported on this methodology both used different baseline BMIs in their calculations. Wei and colleagues [16] combined the data from the ARIC, CARDIA, and Framingham Heart studies to examine the relationship between “BMI-years” and incident diabetes. They followed over 17,000 participants stratified into two baseline age groups (30–44 and 45–59) for a median of 9 years and found that the younger cohort experienced a greater risk for incident diabetes than the older group for a given increase in BMI-years. However, unlike the other three studies, they used each subject's baseline BMI as the reference BMI and were once again limited by the short study duration and absence of early life data. Interestingly, another report (2015) [23] that analyzed the data from the CARDIA Study found a significant relationship between “excess BMI-years” (as well as “excess waist circumference-years”) and incident cardiovascular disease. In doing so, they examined other outcomes including diabetes, which was also significantly associated with these measures. Finally, Abdullah and colleagues most recently [17] reported on the relationship between “obese-years” and T2DM risk, again using the data from the Framingham Offspring Study. They followed 5132 participants aged 5–85 (mean baseline age 37) for up to 45 years and, like the other studies, found a strong association between an increase in obese-years and T2DM risk. While study duration was longer than that of the other three studies and some children were included, the mean baseline age of 37 indicates that most participants were adults at the study onset. Moreover, a BMI of 29 was employed as the reference value to subtract from participants' BMIs. If the result was a negative value, it was considered to be 0. Thus, the main limitation to this study was the inability to consider the possible impact of overweight (BMIs 25–29) on diabetes risk.

While the studies summarized above are important in demonstrating a strong relationship between excess weight, its duration, and diabetes risk, we believe that our analysis of the data from the NCDS has methodological advantages compared to the others and is more robust in helping us to understand the contribution of increased adiposity to the pathophysiology of T2DM. With respect to methodology, we have first used a reference BMI of 25 (or corresponding values for children/youth) so we are able to take into account the impact of overweight (as well as obesity) on diabetes risk. Second, when subtracting participants' BMIs below 25 from our BMI reference, we have considered the difference to be zero. Although the studies by Lee et al. and Bouchard et al. also used a BMI reference of 25, participants' BMIs below that value created a negative balance which implies a reduced diabetes risk for which we can find no evidence. Because of these differences, we have used the new term “cumulative obesity dose” to describe our method. Third, our study is the only one to examine the relationship between cumulative obesity dose and diabetes risk in a birth cohort with data covering the time from birth to 50 years of age. It is therefore unique in its ability to include BMI values from childhood and adolescence in its analysis. Fourth, we were able to adjust for other important diabetes predictors including sex and socioeconomic status. Lower social class was a significant diabetes risk factor in univariate analysis but did not remain in final models, possibly because of the relationship between lower socioeconomic status and low birth weight. In a recent meta-analysis of the relationship between birth weight and type 2 diabetes, adjustment for social class did not appreciably influence that relationship [12]. Finally, ours is the only study to incorporate the impact of birth weight in analyzing the relationship between cumulative obesity dose and T2DM.

In the meta-analysis examining the birth weight/T2DM relationship [12], both high birth weight and low birth weight were shown to increase T2DM risk. The association of high birth weight with diabetes was initially demonstrated among indigenous peoples in North America and appears related to the impact of elevated rates of diabetic pregnancies and associated macrosomia in elevating T2DM risk in the offspring of diabetic mothers [22]. In contrast, low birth weight has been most commonly implicated as a T2DM risk factor among populations of middle-aged and older Europeans. Hales and Barker have proposed that this linkage is initiated through fetal undernutrition leading to a “thrifty phenotype” [24] wherein a maladaptive response to impaired islet cell development contributes to the later development of insulin resistance. However, it is not clear from previous studies whether the influence of low birth weight on the later development of T2DM is mediated through obesity or some other factors. The meta-analysis above found that the low birth weight/T2DM relationship was only minimally strengthened by adjustment for the current BMI. In the more recent Black Women's Health Study [25], low birth weight was also associated with increased T2DM risk but once again, adjustment for the current BMI did not materially change the incidence risk ratios.

As far as we are aware, no one has previously examined the possible interaction between birth weight and any measure of obesity history (i.e., rather than the current BMI) in strengthening the risk of T2DM. We have now shown that the relationship between low birth weight and diabetes is modestly strengthened by adjusting for the time-varying BMI and last BMI (AHR increased from 1.444 without measures of obesity to 1.511 and 1.522 for the time-varying BMI and last BMI, resp.). However, after adjusting for cumulative obesity dose, the AHR for the relationship between low birth weight and diabetes increased substantively from 1.444 to 1.686 and became significant (*P* = 0.0113). Thus, it appears that the degree and duration of excess weight do indeed play an intermediate role in the link between low birth weight and T2DM. Whether this is because the metabolic impact of the “thrifty phenotype” contributes to the predisposition to the early development of obesity and the subsequent increase in diabetes risk or the link between low birth weight and diabetes is simply enhanced by an unrelated and prolonged exposure to increased adiposity is not clear from our study. However, as far as we are aware, our findings provide the strongest evidence to date for an intermediary role of obesity in the link between low birth weight and diabetes and this has been made possible by considering obesity history rather than the current BMI.

We have summarized the strengths of our study above. Limitations include the large 82.4% loss to follow-up (although this is not unexpected in a cohort followed for 50 years) and the homogeneity of a white British population which prevented us from examining the possible impact of ethnicity on diabetes risk. We were unable to differentiate between type 1 and type 2 diabetes, but given that over 90% of incident cases of diabetes occurring during adulthood are T2DM, we believe that our findings largely relate to the impact of cumulative obesity dose on T2DM risk.

## 5. Conclusions

Cumulative obesity dose is an improved method for evaluating the impact of obesity degree and duration on diabetes risk and provides a better predictive ability for determining diabetes risk than either the time-varying BMI or current BMI. This supports the concept that it is the prolonged exposure to the metabolic consequences of excess weight that confers its pathophysiological link with T2DM, and this is consistent with an adverse effect on beta cell function and insulin resistance. Moreover, we have now made the unique observation that the link between low birth weight and T2DM is strengthened by an elevation in cumulative obesity dose, suggesting that this link is either mediated through or amplified by an early and sustained exposure to obesity.

## Figures and Tables

**Table 1:** Study participants by sweep, age, sex, diabetes status, proportion of elevated BMI, and loss to follow-up.

Sweep	Age	Male				Female				All				Loss to follow-up
		Diabetes-free	Newly diagnosed diabetes	Total	Elevated BMI (*N* (%))	Diabetes-free	Newly diagnosed diabetes	Total	Elevated BMI (*N* (%))	Diabetes-free	Newly diagnosed diabetes	Total	Elevated BMI (*N* (%))	
1	7	8537	2	8539	613 (7.18%)	8030	2	8032	770 (9.59%)	16,567	4	16,571	1383 (8.35%)	2
2	11	7683	6	7689	504 (6.55%)	7381	0	7381	688 (9.32%)	15,064	6	15,070	1192 (7.91%)	1503
3	16	7674	14	7688	449 (5.84%)	7374	9	7383	602 (8.15%)	15,048	23	15,071	1051 (6.97%)	1502
4	23	5297	5	5302	1006 (18.97%)	5495	10	5505	800 (14.53%)	10,792	15	10,807	1806 (16.71%)	5766
5	33	5571	18	5589	2753 (49.26%)	5766	27	5793	1985 (34.27%)	11,337	45	11,382	4738 (41.63%)	5191
6	42	5498	61	5559	3477 (62.55%)	5690	41	5731	2503 (43.67%)	11,188	102	11,290	5980 (52.97%)	5283
7	46	1191	76	1267	896 (70.72%)	1392	48	1440	765 (53.13%)	2583	124	2707	1661 (61.36%)	13,866
8	50	1319	29	1348	988 (73.29%)	1545	25	1570	923 (58.79%)	2864	54	2918	1911 (65.49%)	13,655
Total			211				162				373			

Elevated BMI (*N* (%)): number and proportion of subjects with BMI above normal (including overweight and obese).

**Table 2:** Unadjusted hazard ratios (HRs) of developing diabetes using univariate Cox regression analyses.

Characteristic	Levels	*N* (%)	Unadjusted HR (95% CI)	*P* value
Ethnicity (*n* = 11316)	Others	251 (2.22)	Reference	
	White	11,065 (97.78)	0.575 (0.306, 1.081)	0.0856

Sex (*n* = 16573)	Females	8034 (48.48)	Reference	
	Males	8539 (51.52)	1.389 (1.131, 1.704)	0.0017^∗^

Birth weight (*n* = 15043)	Normal	12,761 (84.83)	Reference	
	Low birth weight	879 (5.84)	1.408 (0.942, 2.104)	0.0948
	High birth weight	1403 (9.33)	1.138 (0.801, 1.618)	0.4695

Social class (*n* = 14764)	I (professional occupations)	654 (4.43)	Reference	
	II (managerial and technical occupations)	1934 (13.10)	1.332 (0.639, 2.777)	0.4446
	III (skilled occupations)	8943 (60.57)	1.963 (1.007, 3.825)	0.0476^∗^
	IV (partly skilled occupations)	1809 (12.25)	2.159 (1.058, 4.406)	0.0344^∗^
	V (unskilled occupations)	1424 (9.65)	2.543 (1.227, 5.271)	0.0121^∗^

Cumulative obesity dose (*n* = 16573)	10-unit increase		1.076 (1.068, 1.084)	<0.0001^∗^

Time-varying BMI (*n* = 16573)	1-unit increase		1.098 (1.081, 1.115)	<0.0001^∗^

Last BMI (*n* = 16573)	1-unit increase		1.137 (1.078, 1.198)	<0.0001^∗^

∗indicates significance at 0.05 level.

**Table 3:** Adjusted hazard ratios (AHRs) of developing diabetes using multivariate Cox regression analyses.

Characteristic	Model number 0		Model number 1		Model number 2		Model number 3	
	AHR (95% CI)	*P* value	AHR (95% CI)	*P* value	AHR (95% CI)	*P* value	AHR (95% CI)	*P* value
*Sex*								
Males versus females	1.328 (1.072, 1.646)	0.0095	1.483 (1.192, 1.843)	0.0004	1.297 (1.046, 1.608)	0.0177	1.329 (1.072, 1.647)	0.0094
*Birth weight*								
Low birth weight versus normal	1.444 (0.966, 2.158)	0.0732	1.686 (1.125, 2.526)	0.0113	1.511 (1.010, 2.260)	0.0444	1.522 (1.017, 2.277)	0.0410
High birth weight versus normal	1.101 (0.774, 1.567)	0.5920	0.922 (0.647, 1.315)	0.6548	1.013 (0.712, 1.442)	0.9422	1.024 (0.719, 1.459)	0.8947
*Cumulative obesity dose*			1.080 (1.071, 1.088)	<0.0001				
*Time-varying BMI*					1.098 (1.080, 1.117)	<0.0001		
*Last BMI*							1.146 (1.084, 1.212)	<0.0001
*Model evaluation*								
AIC	5940.762		5716.317		5851.199		5922.872	
BIC	5952.257		5731.645		5866.526		5938.200	

Model number 1 included sex, birth weight, and cumulative obesity dose as covariates; model number 2 includes sex, birth weight, and time-varying BMI as covariates; model number 3 includes sex, birth weight, and last BMI as covariates.

**Table 4:** Adjusted hazard ratios (AHRs) of developing diabetes using multivariate Cox regression analyses: participants with the overweight/obesity onset during follow-up.

Characteristic	Model number 1		Model number 2		Model number 3	
	AHR (95% CI)	*P* value	AHR (95% CI)	*P* value	AHR (95% CI)	*P* value
*Sex*						
Males versus females	1.544 (1.211, 1.968)	0.0004	1.399 (1.101, 1.777)	0.0060	1.405 (1.106, 1.784)	0.0053
*Birth weight*						
Low birth weight versus normal	1.783 (1.137, 2.798)	0.0118	1.593 (1.017, 2.493)	0.0418	1.565 (1.000, 2.450)	0.0410
High birth weight versus normal	0.946 (0.651, 1.375)	0.7713	1.028 (0.708, 1.491)	0.8853	1.060 (0.731, 1.537)	0.8947
*Age of obesity onset*	0.988 (0.977, 1.000)	0.0434	0.976 (0.965, 0.986)	<0.0001	0.973 (0.962, 0.983)	<0.0001
*Cumulative obesity dose*	1.072 (1.062, 1.081)	<0.0001				
*Time-varying BMI*			1.080 (1.059, 1.101)	<0.0001		
*Last BMI*					1.044 (1.022, 1.066)	<0.0001
*Model comparison*						
Model with age of obesity onset						
AIC	4473.633		4605.563		4639.406	
BIC	4491.824		4623.772		4657.616	
Model without age of obesity onset						
AIC	4475.874		4625.230		4666.389	
BIC	4490.427		4639.798		4680.957	

**Table 5:** Interaction between cumulative obesity dose and age groups in risk for diabetes.

Characteristic	AHR (95% CI)	*P* value
*Sex*		
Males versus females	1.480 (1.190, 1.839)	0.0004
*Birth weight*		
Low birth weight versus normal	1.684 (1.124, 2.522)	0.0115
High birth weight versus normal	0.914 (0.641, 1.304)	0.6209
*Cumulative obesity dose*		
Childhood (age 7–16)	1.187 (0.877, 1.607)	0.2678
Young adult (age 23–42)	1.097 (1.081, 1.113)	<0.0001
Older adult (age above 42)	1.074 (1.064, 1.084)	<0.0001
*Model evaluation*		
AIC	5714.463	
BIC	5737.455	

## Supplementary Materials

Supplementary Materials Appendix A provides a detailed description of the time-varying BMI including formulae.
