# Supplementary material for: Low Birth Weight, Cumulative Obesity Dose, and the Risk of Incident Type 2 Diabetes
Source: J Diabetes Res. 2018 Jan 9;2018:8435762. doi: 10.1155/2018/8435762 (PMC5818910; doi:10.1155/2018/8435762)
Supplement: Supplementary Materials — Appendix A provides a detailed description of the time-varying BMI including formulae. [file 8435762.f1.pdf]

Appendix A: Model Description of Time Varying BMI

(Cox proportional hazards model with time-dependent explanatory variables)

Reference: Modelling Survival Data in Medical Research (Third Edition, 2015) by David Collett, Chapman & Hall

Below describes the Cox proportional hazards model with a time-dependent explanatory variable, where a 'time-dependent' or 'time-varying' factor refers to a series of measurements of that risk factor during follow-up.

Let T be the time to diabetes and let Z represent a time dependent covariate (i.e. time varying BMI or cumulative obesity dose). We use $Z(t)$ to denote the value of Z at time t, and $\bar{Z}(t) = \{Z(s): 0 \leq s \leq t\}$ to denote the history of the covariates up to time t. It is convenient to formulate the effects of covariates on the failure time through the hazard function. The conditional hazard function of T given $\bar{Z}(t)$ is

$$h\big(t \big| \bar{Z}(t)\big) = \lim_{\Delta t \to 0} \frac{p(t \leq T \leq t + \Delta t | T \geq t, \bar{Z}(t)\ )}{\Delta t}$$

where the numerator of this expression is the conditional probability that the event will occur in the interval $[t, t + \Delta t)$ given those who are at risk of getting diabetes at time t and the denominator is the width of the interval. Taking the limit as the width of the interval goes to zero, the hazard function is the instantaneous rate of diabetes occurrence.

The Cox proportional hazard model is then specified as,

$$h\big(t \big| \bar{Z}(t)\big) = h_0(t) e^{\beta Z(t)}$$

where the baseline hazard function, $h_0(t)$, is interpreted as the hazard function for an individual when all the variables are zero at the time origin. Here, β denotes the regression coefficient for $Z(t)$. To provide an interpretation of βin this model, we take the ratio of the hazard functions at time t for two individuals, the rth and sth, say. The relative ratio is given by

$$\frac{h_r(t \,|\, \overline{Z_r}(t)\ )}{h_s(t \,|\, \overline{Z_s}(t)\ )} = e^{\beta [Z_r(t) - Z_s(t)]}$$

The regression coefficient can be interpreted as the log hazard ratio for two individuals whose value of the time varying covariate at a given time t differ by one unit with two individuals having the same values of all the other variables at that time.

When the Cox regression model is extended to incorporate time dependent variable, the partial likelihood can be generalized from the conventional Cox regression. Suppose there are n individuals in the study, among whom there are r distinct event times and n-r right censored survival times. Let $t_{(1)} < t_{(2)} < \cdots < t_{(r)}$, where $t_{(j)}$ is the jth ordered event time. The set of individuals at risk (risk set) at time $t_{(j)}$ is denoted as $R(t_{(j)})$, which is a group of individuals who are alive and uncensored at a time just prior to $t_{(j)}$. The partial likelihood can be then written as:

$$L(\beta) = \prod_{j=1}^{r} \frac{e^{\beta Z(t_{(j)})}}{\sum_{l \in R(t_{(j)})} e^{\beta Z(t_l)}}$$

The maximum likelihood estimates of β can be found by maximizing this log partial likelihood function using numerical methods.

To comprehend the concept of Cox regression with time varying covariates in a more straightforward way, the follow-up time for each patient is divided into different time windows according to the unique event times. For each time window, a separate Cox analysis is carried out using the time-dependent variable at the beginning of that specific time window. Then, a weighted average of all the time window-specific results is calculated as the estimated relative ratio.
